# Retraction: Combination effect of cold atmospheric plasma with green synthesized zero-valent iron nanoparticles in the treatment of melanoma cancer model

**DOI:** 10.1371/journal.pone.0304502

**Published:** 2024-05-23

**Authors:** The PLOS ONE Editors

After this article [1] was published, concerns were raised about Figs 4A and 3A.

Specifically:

- The Fig 3A L929 CAP panel of [1] appears similar to the Fig 3A L929 Control panel of [2] when rotated 180 degrees and to the Fig 1 Untreated L929 panel of [3] when rotated 180 degrees.
- In Fig 4A, the left side of the L929 Untreated panel appears similar to the upper left side of the L929 CAP+nZVI when flipped vertically and rotated 180 degrees.
- In Fig 4A, the left side of the B16-F10 CAP panel appears similar to the lower left side of the B16-F10 nZVI panel when rotated 90 degrees.

The corresponding author acknowledged that errors occurred in the preparation of Figs 3 and 4, and provided underlying data. Upon editorial assessment, additional concerns were identified in these data, and therefore, the concerns described above remain unresolved.

In light of these issues, which raise concerns about the reliability and integrity of the published results, the *PLOS ONE* Editors retract this article.

All authors did not agree with the retraction.
